# Preterm birth and exercise capacity: what do we currently know?

**DOI:** 10.3389/fped.2023.1222731

**Published:** 2023-10-06

**Authors:** Christopher A. O’Dea, Michael L. Beaven, Andrew C. Wilson, Elizabeth F. Smith, Andrew Maiorana, Shannon J. Simpson

**Affiliations:** ^1^ Respiratory Medicine, Perth Children’s Hospital, Perth, WA, Australia; ^2^ Wal-yan Respiratory Research Centre, Telethon Kids Institute, Perth, WA, Australia; ^3^ School of Allied Health, Curtin University, Perth, WA, Australia; ^4^ Department of Allied Health, Fiona Stanley Hospital, Perth, WA, Australia

**Keywords:** exercise and lung disease, bronchopulmonary dysplasia, pediatric lung disease, lung physiology, pediatric exercise physiology

## Abstract

**Objectives:**

The long-term cardiopulmonary outcomes following preterm birth during the surfactant era remain unclear. Respiratory symptoms, particularly exertional symptoms, are common in preterm children. Therefore, cardiopulmonary exercise testing may provide insights into the pathophysiology driving exertional respiratory symptoms in those born preterm. This review aims to outline the current knowledge of cardiopulmonary exercise testing in the assessment of children born preterm in the surfactant era.

**Design:**

This study is a narrative literature review.

**Methods:**

Published manuscripts concerning the assessment of pulmonary outcomes using cardiopulmonary exercise testing in preterm children (aged <18 years) were reviewed. Search terms related to preterm birth, bronchopulmonary dysplasia, and exercise were entered into electronic databases, including Medline, PubMed, and Google Scholar. Reference lists from included studies were scanned for additional manuscripts.

**Results:**

Preterm children have disrupted lung development with significant structural and functional lung disease and increased respiratory symptoms. The association between these (resting) assessments of respiratory health and exercise capacity is unclear; however, expiratory flow limitation and an altered ventilatory response (rapid, shallow breathing) are seen during exercise. Due to the heterogeneity of participants, treatments, and exercise protocols, the effect of the aforementioned limitations on exercise capacity in children born preterm is conflicting and poorly understood.

**Conclusion:**

Risk factors for reduced exercise capacity in those born preterm remain poorly understood; however, utilizing cardiopulmonary exercise testing to its full potential, the pathophysiology of exercise limitation in survivors of preterm birth will enhance our understanding of the role exercise may play. The role of exercise interventions in mitigating the risk of chronic disease and premature death following preterm birth has yet to be fully realized and should be a focus of future robust randomized controlled trials.

## Introduction

Preterm births [less than 37 weeks completed gestational age (GA)] make up approximately 11% of births worldwide (1), with an increased rate of children born preterm reported from 1990 to 2010 (1). Babies surviving preterm birth are at risk of significant health challenges throughout life, including chronic respiratory, neurodevelopmental, and cardiovascular disorders. Long-term health challenges following preterm birth are accentuated in those with bronchopulmonary dysplasia (BPD), a chronic lung disease resulting from the disruption of development and subsequent treatment-induced injury (2). Advancements in neonatal care in the 1990s improved the survival of infants at borderline viable gestational ages, resulting in a change in the pulmonary pathology associated with preterm birth and BPD from the condition first described in the 1960s (3). The hallmark characteristics of contemporary BPD include immature airways, arteries and veins, few or no alveoli, and an inefficient gas exchange area (3). Respiratory health assessments conducted in childhood (4) and young adulthood (5, 6) reveal reduced lung function and structural abnormalities in modern survivors of preterm birth, raising concerns for early-onset adult respiratory disease, including chronic obstructive pulmonary disease (COPD) (7). While the long-term respiratory consequences of preterm birth will likely not be fully realized until the fourth, fifth, and sixth decades of life, recent evidence suggests that infants born preterm in the contemporary era are at risk of developing cardiometabolic diseases in adulthood (8) and increased respiratory morbidity and exertional dyspnea (4, 9, 10). Cardiopulmonary exercise testing (CPET) provides insights into the etiology of cardiopulmonary function following preterm birth and has the potential to identify targets for therapeutic interventions. This narrative review aims to summarize the current knowledge of cardiopulmonary exercise testing in the assessment of pulmonary outcomes in children born preterm in the surfactant era.

## Cardiopulmonary exercise testing

CPET assesses the integrative response of the cardiovascular, pulmonary, and skeletal muscle systems to exercise, providing clinical information that may not be apparent at rest. Indeed, health status correlates more strongly with exercise responses than resting measurements across various different pathologies (11). Performing exercise assessments enables the identification of early disrupted physiology and causes of exercise intolerance.

Children born preterm are at higher risk of cardiometabolic diseases (8), such as increased vascular resistance and blood pressure (12), alterations in fat distribution (13), and impaired glucose regulation (14). Understanding the exercise capacity of children born preterm and the systems limiting exercise capacity improves our understanding of deterrents to partaking in physical activity. Importantly, understanding barriers to exercise participation in this population can inform programs designed to mitigate the health risks associated with preterm birth.

## Peak aerobic exercise capacity (V̇O_2_)

Peak V̇O_2_, a measure of the oxygen uptake of the skeletal muscles, provides a measure of aerobic capacity and is an independent predictor of all-cause mortality (15). Peak V̇O_2_ independently predicts hospitalizations and future exacerbations in other parenchymal lung diseases such as cystic fibrosis (16–19), and hence, its predictive use in children born preterm has been explored. Data on the aerobic capacity of preterm children born in the surfactant era are conflicting: some studies show a lower peak V̇O_2_ (10, 20, 21), whereas others have shown no difference (22–26). Comparisons of these studies are difficult due to the variability within the inclusion criteria of the cohorts studied, such as GA, birth weight, the presence and severity of BPD, and the nature of neonatal care.

### Neonatal factors associated with lower peak V̇O_2_

Our literature review shows that the impact of neonatal factors associated with preterm birth on peak V̇O_2_ is poorly understood. Hochwald et al. reported similar deficits in V̇O_2_ at peak exercise between participants born early preterm (<30 weeks GA) and late preterm (34–36.6 weeks GA) (27). Several studies have reported no association between peak V̇O_2_ and the presence or severity of BPD (26, 28). We have previously reported that after accounting for height, weight, and sex, there were no associations between neonatal factors, structural abnormalities on CT, and lung function for V̇O_2_ at peak exercise (26). Collinearities between gestational age, the severity of BPD, and neonatal interventions, including the duration of mechanical ventilation and supplemental oxygen, make the effect of neonatal histories difficult to elucidate. Consequently, few studies have accurately identified or are able to report the independent effect of gestational age or duration of mechanical ventilation and supplemental oxygen on the aerobic exercise capacity of children born preterm (10, 21, 26).

### Association between lung structure and peak V̇O_2_

Structural lung disease accounts for 42% of the variability in peak V̇O_2_ in other chronic chest diseases, such as cystic fibrosis, and the total CT score is a stronger predictor for exercise limitation than lung function or body mass (29). To date, only one published study has explored the relationship between structural lung abnormalities in preterm children and exercise capacity, showing no association between peak exercise outcomes and structural lung disease. However, this study did not demonstrate significant exercise impairment in children born preterm (26). Given the high prevalence of exertional symptoms and structural lung disease, an association between exercise capacity and structural lung disease is plausible for children born preterm.

## Impact of exercise modality on peak V̇O_2_

Differences in the methods of exercise testing [e.g., cycle ergometers (10, 21, 23–25, 30, 31), treadmills (9, 22, 28), or shuttle run tests (20, 32–35)] likely contribute to the conflicting outcomes for aerobic capacity in this group. Generally, most treadmill exercise test studies report no difference in aerobic capacity (9, 22), while all studies involving shuttle runs and most studies involving cycle ergometers show lower peak V̇O_2_ in the preterm population. The varied methodologies of exercise testing make comparisons difficult due to the different advantages and limitations of each method.

### Shuttle run assessment

The estimation of peak V̇O_2_ from shuttle runs involves physical movements that are familiar to children. However, the shuttle run is demanding from a cognitive perspective and may be challenging for preterm children with impaired neurodevelopmental performance (1). Burns et al. (34) reported that motor impairment was the only predictor of estimated peak V̇O_2_ in extremely low birth weight children, which may suggest that the reductions reported are perhaps due to poor coordination rather than cardiopulmonary limitation. Unfortunately, other factors that may limit exercise during a shuttle run test, such as workload, ventilatory response, or gas exchange during exercise, cannot be assessed, limiting the utility of these studies in understanding the underlying exercise limitation.

### Cycle ergometry

Most exercise studies in children born preterm in the surfactant era used cycle ergometry, with some (10, 21, 31, 36) but not all (23–25, 30) studies showing a reduced peak V̇O_2_. Reductions in workload (10) and V̇O_2_/work (24, 31) were reported in the preterm population, even with normal peak V̇O_2_ (25, 30). These differences in workload and V̇O_2_/work may suggest differences in cardiovascular or peripheral muscle function underlie the reduced exercise capacity, as the V̇O_2_/work slope is related to cardiovascular and musculoskeletal responses to exercise (31, 37). The assessment of peak outcomes in a cycle ergometer test relies on the muscular endurance of the legs, in particular, the quadriceps. It is also plausible that the low workload and V̇O_2_/work relationship may be due to changes in the peripheral muscle mass (31).

Children born preterm have a persistent reduction in body mass with an overall reduction in bone, fat, and fat-free mass; however, there is no difference in the percentage of fat-free mass, suggesting that the overall reduction in body mass is proportional, even when accounting for height (13). This reduction in fat-free mass may lead to a reduction in peripheral muscle strength; indeed, Vardar-Yagli et al. (38) reported a reduction in peripheral muscle strength evaluated using a dynamometer in children born preterm. In particular, Vardar-Yagli et al. reported a significantly greater reduction in lower extremity muscle strength compared to upper body extremities. As exercise using a cycler ergometer requires lower extremity muscle strength, this reduced peripheral strength may make it more difficult for children born preterm to continue cycling at higher workloads, resulting in a reduced peak V̇O_2_ and V̇O_2_/work slope.

### Treadmill

Preterm children born in the post-surfactant era typically have a normal peak V̇O_2_ when assessed using a treadmill-based exercise protocol (9, 22). A sensitivity analysis performed by Edwards et al. comparing metadata from different exercise testing modalities showed that treadmill testing was more likely to identify a difference between term and preterm groups than cycle ergometry (39). However, we note that the data that most heavily contributed to the differences observed were based on an abstract containing preliminary data (40), which, when submitted for full publication, showed no differences between the term and preterm groups (9). Exercise testing using a treadmill is advantageous because it mimics daily activities such as running and walking. The treadmill-based exercise involves the recruitment of a larger muscle mass compared to the predominately lower body recruitment in cycle-based exercise. This results in a higher peak V̇O_2_ and peak heart rate during a treadmill-based CPET than those obtained when using cycle ergometry in healthy subjects (41). Therefore, differences in muscle mass recruitment may explain the contrasting outcomes of the studies using cycle and treadmill protocols. However, treadmill exercise testing also relies on the child's cognitive ability to run to maximal exertion. Preterm children with muscle incoordination or gait impairment may terminate the exercise test early, resulting in a measurement bias favoring healthy children. In addition, preterm children unable to perform a peak exercise test are likelier to have worse lung function and more parentally reported symptoms (9). The literature contains no direct comparisons of treadmill and cycle ergometry feasibility in children born preterm, and testing modality is ultimately determined by equipment availability and the preference of the supervising team.

## Oxygen update efficiency slope

Developmental delay and poor coordination are well-recognized in children following preterm birth. Accurate peak V̇O_2_ measurements can be difficult to achieve in this group as they are critically dependent on subject motivation and coordination and also on the skills of the observer. Baba et al. (42) proposed the oxygen uptake efficiency slope (OUES), a submaximal measurement of cardiopulmonary functional capacity. OUES is derived from the slope of V̇O_2_ plotted against the log of minute ventilation (V̇_E_), which is linear throughout testing,, and is independent of workload, enabling the assessment of cardiopulmonary exercise capacity even from submaximal tests. OUES has been validated against peak V̇O_2_ in children with congenital heart disease when assessed at the ventilatory threshold, 75% or 100% of peak V̇O_2_. Further, OUES has been proven to differentiate between disease groups (43, 44). The utility of OUES in a preterm population is largely unstudied. Therefore, it should be an area of future research, given the variability in reported outcomes, the difficulties testing in this population, and the possibility of healthy survivor bias. Hence, the wider use of OUES in the assessment of aerobic capacity in young children would allow for the generalization of results to a broader preterm population.

## Ventilatory response to exercise

Exertional dyspnea in preterm-born individuals is attributed to an altered ventilatory response to exercise, which may be associated with decreased lung function. A growing body of evidence suggests that children born preterm mount an altered ventilatory response to exercise, characterized by a rapid breathing pattern (9, 10, 24, 25, 31). Some studies also report a shallow breathing pattern at peak exercise, identified by a reduced tidal volume (10, 21, 24, 31). Supporting these findings, our group (26) reported significantly higher breathing frequency to tidal volume ratios at peak exercise in school-aged children born very preterm with BPD. Two studies on children delivered extremely preterm (10, 21) showed a reduction in peak V̇_E_; however, studies on children delivered very preterm showed no alterations (9, 23, 31). The differences in results between children born extremely preterm and very preterm suggest that gestational age and lung development may play a role in altered ventilatory response.

Ventilatory efficiency during exercise as assessed by ventilatory equivalents for carbon dioxide and oxygen (V̇_E_/V̇CO_2_ and V̇_E_/V̇O_2_, respectively) independently predict hospitalizations and future exacerbations in other parenchymal lung diseases such as cystic fibrosis (16–19); ventilatory efficiency can be assessed using the V̇_E_/V̇CO_2_ slope. The effect of preterm birth on ventilatory efficiency is unresolved: two studies (21, 25) showed an increased V̇_E_/V̇CO_2_ slope, suggesting inefficient ventilation, while a further study showed no difference (31). In a study on very low birth weight (VLBW) children, conducted by Rideau Batista Novais et al. (25), the V̇_E_/V̇CO_2_ was elevated and associated with a reduction in end-tidal CO_2_, suggesting inefficient ventilation and hyperventilation during exercise (45). However, peak exercise ventilatory indices were similar between the VLBW and control groups. The authors hypothesized that this finding was due to either an altered CO_2_ setpoint or inspiratory muscle fatigue. However, the plausibility of a significant alteration in CO_2_ setpoint is unclear, given the normal peak exercise ventilatory outcomes. Respiratory muscle fatigue was suggested to be a more likely contributor to the altered ventilatory response, given increased inspiratory load for the same exercise intensity and, therefore, likely an increased susceptibility to fatigue (25). Supporting this hypothesis, Davidson et al. (46) found elevated sternocleidomastoid muscle activity in children born preterm, suggesting increased respiratory muscle activity at high-intensity exercise, which may lead to respiratory muscle fatigue. However, the mechanism(s) driving respiratory muscle fatigue remains largely unknown.

## Expiratory flow limitation

One factor potentially contributing to altered ventilatory responses during exercise may be expiratory flow limitation (EFL). EFL is associated with an impaired ventilatory response to exercise in asthma, cystic fibrosis, and COPD (47–50). We identified two studies that assessed the prevalence and impact of EFL on exercise in preterm children born in the surfactant era (9, 21). Both studies showed that approximately half of all preterm children develop significant EFL during exercise compared to 25%–30% of term-born controls. Contrary to other obstructive lung diseases, the inspiratory capacity during exercise and the operational lung volumes were not altered in those born preterm, suggesting that gas trapping and airway collapse are not likely causes for EFL in this population. Instead, increased EFL without changes in operating lung volumes may be due to reduced pulmonary compliance and the consequent increased elastic load of breathing, respiratory muscle weakness, and altered respiratory mechanics. Preterm children may have respiratory muscle weakness, which may lead to an inability to overcome the increased work of breathing, leading to EFL; this increased work of breathing may also lead to dyspnea. However, there is no association between EFL and alterations in exercise capacity or ventilatory response to exercise (9, 21). In a 2018 study, we showed that the presence of EFL is associated with a lower gestational age and reduced FEV_1_/FVC (9). The influence of low FEV_1_/FVC is supported by the findings that EFL in healthy adults is explained mainly by alterations in lung and airway size or dysanapsis (smaller airway size relative to lung volume), which limits the capacity to generate the flows and volumes required during exercise (51). Given the disrupted lung growth and development associated with preterm birth, airway dysanapsis likely contributes to EFL in conjunction with respiratory muscle weakness and altered respiratory mechanics. Understanding the mechanisms underlying EFL and their contribution to exercise limitation will be key to clarifying the mechanisms of the altered ventilatory response and identifying potential therapeutic options for reducing respiratory morbidity.

## Early V̇O_2_ recovery

Exertional dyspnea in preterm children is still poorly understood; there is no obvious correlation between exertional dyspnea with exercise outcomes despite reduced lung function (9, 10). Dyspnea is related to respiratory muscle strength and endurance in adults with congestive heart failure, and respiratory muscle strength is related to early recovery oxygen kinetics (52). Early recovery following maximal exercise is characterized by rapid payback of oxygen debt and resynthesis of phosphocreatine. This process depends on the transport and utilization of oxygen (53). In children with chronic chest diseases, such as cystic fibrosis and bronchiectasis, the V̇O_2_ recovery following maximal exercise is delayed (measured by the time to return to 50% of peak V̇O_2_) and is associated with disease severity. However, there is a large variability in the V̇O_2_ recovery rate (53). Recovery following a maximal exercise test depends not only on the size of the oxygen debt (larger debt, faster recovery) but also on the ability of the cardiovascular system to transport oxygen. Therefore, recovery may be a measure of cardiovascular fitness (54) or the ability of the skeletal muscles to take up the delivered oxygen due to alterations to structure or function (55). Currently, there is no evidence regarding V̇O_2_ recovery in children born preterm. However, assessment of early V̇O_2_ recovery may identify differences given the altered ventilatory response in preterm children (which may be related to altered respiratory muscle strength) and could help explain increased exertional symptoms.

## Heart rate recovery

Following maximal exercise, the immediate heart rate recovery is mediated by the autonomic nervous system with activation of the parasympathetic nervous system via the reactivation of the vagal nervous system and the withdrawal of the sympathetic nervous system. Reduced vagal activity, which may delay heart rate recovery, is a predictor of all-cause mortality and is associated with metabolic risk factors in healthy children (56). The development of the autonomic nervous system occurs in the third trimester after the delivery of most preterm infants, making autonomic nervous system impairment plausible (57). To date, limited research has reported heart rate recovery following peak exercise in survivors of preterm birth. One study by Haraldsdottir et al. assessed 12 preterm patients who reported delayed heart rate recovery in adult survivors of preterm birth; however, it did not investigate the impact of neonatal variables (57). Another study by Huckstep et al. found that young adults who were born moderately preterm have lower peak V̇O_2_ and slower heart rate recovery following exercise compared to term controls. They concluded that impaired myocardial functional reserve may be a key factor underpinning these impairments (36). Given the known risks for metabolic disease in preterm children, altered heart rate recovery in this population may justify the evaluation of a cardiopulmonary rehabilitation program to help modify these risks and reduce exertional dyspnea. This has been successful in other childhood diseases; cardiac rehabilitation was shown to improve heart rate recovery in children with complex congenital heart disease (58). The role of cardiopulmonary rehabilitation programs in improving heart rate recovery and other exercise outcomes in those born preterm is yet to be fully realized; however, it should be a focus of future robust randomized controlled trials.

## Conclusion

Children born preterm have disrupted lung growth and development, which results in significant respiratory symptoms, structural changes to the lung, and a reduction in lung function. During exercise, a significantly altered ventilatory response is evident; however, the underlying mechanisms and their clinical significance remain unclear. Associations between resting assessments of lung health (function, structure, and symptoms) and exercise limitation are poorly understood and cannot accurately predict exercise limitation. Due to the heterogeneity of participants, treatments, and exercise protocols, there are variable reports in the literature about the nature and extent of exercise limitation in those born preterm.

CPET assesses interactions between the metabolic, respiratory, cardiovascular, and musculoskeletal systems, and by utilizing this tool to its full potential, the pathophysiological consequences of preterm birth can be further investigated. Enhanced knowledge of the pathophysiology of impaired lung function in childhood will open new avenues for interventions to prevent further deterioration in lung function throughout childhood.
